# Opportunity for verbalization does not improve visual change detection performance: A state-trace analysis

**DOI:** 10.3758/s13428-016-0741-1

**Published:** 2016-06-10

**Authors:** Florian Sense, Candice C. Morey, Melissa Prince, Andrew Heathcote, Richard D. Morey

**Affiliations:** 1 Department of Experimental Psychology & Department of Psychometrics and Statistics, University of Groningen, Groningen, Netherlands; 2 Department of Psychology, University of Edinburgh, Edinburgh, UK; 3 School of Psychology, Flinders University, Bedford Park, Australia; 4 School of Medicine, The University of Tasmania, Hobart, Australia; 5 School of Psychology, Cardiff University, Cardiff, UK

**Keywords:** Articulatory suppression, Change detection, State-trace, Working memory capacity, Verbalization

## Abstract

Evidence suggests that there is a tendency to verbally recode visually-presented information, and that in some cases verbal recoding can boost memory performance. According to multi-component models of working memory, memory performance is increased because task-relevant information is simultaneously maintained in two codes. The possibility of dual encoding is problematic if the goal is to measure capacity for visual information exclusively. To counteract this possibility, articulatory suppression is frequently used with visual change detection tasks specifically to prevent verbalization of visual stimuli. But is this precaution always necessary? There is little reason to believe that concurrent articulation affects performance in typical visual change detection tasks, suggesting that verbal recoding might not be likely to occur in this paradigm, and if not, precautionary articulatory suppression would not always be necessary. We present evidence confirming that articulatory suppression has no discernible effect on performance in a typical visual change-detection task in which abstract patterns are briefly presented. A comprehensive analysis using both descriptive statistics and Bayesian state-trace analysis revealed no evidence for any complex relationship between articulatory suppression and performance that would be consistent with a verbal recoding explanation. Instead, the evidence favors the simpler explanation that verbal strategies were either not deployed in the task or, if they were, were not effective in improving performance, and thus have no influence on visual working memory as measured during visual change detection. We conclude that in visual change detection experiments in which abstract visual stimuli are briefly presented, pre-cautionary articulatory suppression is unnecessary.

During his seminal experiments on human memory, Sperling noticed that many of his participants verbalized and repeated to-be-remembered material during retention, even if the studied material was not aurally presented. Sperling (1967) pointed out that visual information can be verbalized and many people reported doing so. This reflection confirmed intuitions that regardless of presentation modality, information may be encoded with some flexibility of representation: visual materials might be maintained in a verbal code, and imagery corresponding to verbal input may likewise become active.

However, demonstrating that recoding can occur does not imply that it always occurs, nor that it is beneficial. Murray (1965) showed that saying visually-presented verbal stimuli out loud improves recall performance relative to mouthing them silently. However, this relationship only seems to persist if the visually-presented material can be verbalized effectively (e.g., verbal stimuli, nameable visual images). The idea that it is the opportunity to rehearse these verbal codes that improves performance also remains a matter for debate, even for serially-ordered verbal stimuli (Lewandowsky and Oberauer 2015). Attempts to verbalize stimuli that are difficult to describe succinctly and accurately (e.g., faces) might actually harm performance (Schooler and Engstler-Schooler 1990). Brandimonte et al. (1992) showed that verbal recoding can be detrimental to a subsequent mental rotation task when the remembered verbal label is not relevant or helpful. What such experiments suggest is that there is a strong tendency to verbally recode visually-presented information, and that in some cases verbal recoding may boost memory performance. This logic is consistent with multi-component models of working memory, which propose that separate short-term memory stores for phonological and visual information can be applied to a short-term memory task (Baddeley, 1986). Naturally, if task-relevant information can be maintained simultaneously in two useful codes, one would expect memory performance to improve.

The possibility of dual encoding is problematic though if the goal is to measure capacity for visual information exclusively. Levy (1971) suggested a method of preventing such recoding via meaningless concurrent articulation. By repeating irrelevant syllables out loud during presentation and retention of visual information, participants’ ability to verbally recode visually-presented stimuli is restricted. This procedure is known as *articulatory suppression* and is commonly used alongside visual change detection tasks with the specifically-stated intention that it is meant to prevent verbalization of visual stimuli (e.g. Allen, Baddeley, & Hitch, 2006; Brockmole, Parra, Sala, & Logie, 2008; Delvenne & Bruyer, 2004; Hollingworth & Rasmussen, 2010; Logie, Brockmole, & Vandenbroucke, 2009; Makovski & Jiang, 2008; Makovski, Sussman, & Jiang, 2008; Matsukura & Hollingworth, 2011; Treisman & Zhang, 2006; van Lamsweerde & Beck, 2012; Woodman & Vogel, 2005, 2008). This precaution is undertaken to ensure that task performance reflects visual memory, rather than some combination of memory for visual images and verbal codes.

The use of precautionary articulatory suppression is common practice despite evidence that articulatory suppression has not been shown to have a measurable effect on some visual change detection tasks (Luria, Sessa, Gotler, Jolicoeur, & Dell’Acqua, 2010; Mate, Allen, & Baqués, 2012; Morey & Cowan, 2004, 2005), nor have small verbal memory loads (Vogel, Woodman, & Luck, 2001). These studies imply that the precaution of employing articulatory suppression may be unnecessary: participants performed no better without articulatory suppression than with it, suggesting that verbal recoding is not the default strategy for visual change detection tasks as typically administered. However, these findings simply report null effects of meaningless articulatory suppression on visual memory tasks, and therefore cannot be taken as strong evidence of the absence of some effect, given sufficient power to detect it. Until a stronger case against verbal recoding during visual change detection can be made, enforcing articulatory suppression to prevent verbalization of visual images is a reasonable way for researchers to better ensure that their measure of visual memory performance is pure. However, enforcing articulation adds a substantial burden to an experiment from both the participant’s and the experimenter’s point of view. If a strong case could be made that possible verbal recoding of visual memoranda does not affect visual memory performance, researchers would be free to forgo including articulatory suppression from some designs.

We report evidence suggesting that articulatory suppression has no discernible effect on performance in a typical visual change-detection task. The experiment was designed so that some change-detection conditions encouraged verbalization by presenting memoranda one at a time. In all cases, the stimuli were arrays of distinctly-colored squares, and the object was to remember the location of each color. We manipulated the number of items in each array, whether the squares were presented simultaneously or sequentially, and whether participants performed articulatory suppression or not. If participants tend to verbally label the stimuli, and if verbal labeling assists the recognition decision, we would expect to observe at least a small benefit of silence over articulation in all conditions. It may also be the case that participants strategically choose when to verbally recode stimuli. If so, we would expect to see selective impairments with articulation for sequentially-presented items, perhaps most strongly for small set sizes where naming all the items might have occurred. In order to discern between small effects of articulation and the null hypothesis of no effect at all, we employ two modes of analysis: first, we provide a straightforward analysis based on descriptive statistics that shows that the effects tend to go in the reverse direction to what is predicted, ruling out evidence for the predicted effect; and second, we employed Bayesian state-trace analysis to show that participants show data patterns more consistent with a single-parameter explanation (visual short term memory) than a more complicated explanation (visual short term memory plus verbal short term memory).

## Methods

Participants performed a visual array change detection task under four conditions formed by the cross of two presentation conditions (sequential and simultaneous) and two articulation conditions (silent and articulatory suppression). The simultaneous presentation condition was the same as a standard visual array change detection task; in the sequential condition, the stimuli were presented one after another. We assumed that presenting visual stimuli sequentially would afford a better opportunity to engage in verbalization, if such verbalization occurs (but see Woodman, Vogel, & Luck, 2012). Articulatory suppression is supposed to prevent participants from employing subvocal verbalization. The combination of simultaneous/sequential and silent/articulate conditions creates combinations of conditions that discourage participants from recruiting verbal resources (i.e. articulate, simultaneous trials) as well as those that make it more likely they could benefit from verbalization (i.e. silent, sequential trials).

### Participants

Fifteen participants (8 female) between the age of 21 and 31 (*M* = 25.4, *SD* = 2.67) were recruited from the population of Groningen. Participants were paid €10 per 90-minute session and recruited through a local online social media group.

All participants were pre-screened for colorblindness and medication use that might affect their cognitive abilities, and all participants reported normal or corrected-to-normal vision and normal hearing. Furthermore, participants were only invited for subsequent sessions if they scored at least 85 % correct on set-size-two trials (across all conditions) in the first session. This cut-off value was chosen based on an unpublished pilot study in which 14 out of the 15 pilot participants performed above 85 %, and the remaining low-performing participant scored near chance (50 %) and was assumed to have ignored the instructions. All fifteen participants in our final sample met this criterion. One of these participants completed only four sessions due to scheduling difficulties, while the remainder of the final sample completed five sessions.

#### Apparatus and Stimuli

The experiment was conducted using MATLAB (2011) using the Psychophysics Toolbox extensions (Brainard 1997; Kleiner et al. 2007; Pelli 1997). The stimuli were colored squares approximately 0.65^∘^×0.65^∘^ presented within a 7.3^∘^×9.8^∘^ area around the screen’s center. On each trial, the colors were randomly sampled without replacement from a set of nine easily-discriminable colors and presented on a gray background. The set of possible colors was identical to the one used by Rouder et al. (2008) with the exception that black was excluded, since Morey (2011) showed that black exhibited markedly different effects in a similar change detection task. Stimuli were shown against a neutral gray background. The items within a single array were always arranged with a minimum distance of 2^∘^ from one another and participants sat approximately 50 cm from the monitors. This setup allowed them to see the entire display without moving their heads.

Feedback was given via one of three clearly-discriminable sounds signaling a correct, incorrect, or invalid response (i.e., a key that was not assigned to either of the two valid responses). The sounds were played through headphones worn throughout the entire experiment.

#### Procedure

Within each session, participants completed one block of trials in which subvocal articulation was suppressed by requiring them to repeat aloud the syllables “ta” and “da” (*articulation* block) and one block in which no such articulatory suppression was enforced (*silent* block). Both the articulation and the silent blocks were further sub-divided in two blocks: one in which stimuli were presented simultaneously and one in which they were presented sequentially. The order in which blocks were completed was determined based on the participants’ IDs and identical in each session. There were 504 trials in each session, yielding a total of 2,520 trials per participant (except for participant 10 who came in for four sessions, contributing 2,016 instead of 2,520 trials).

**Fig. 1:**
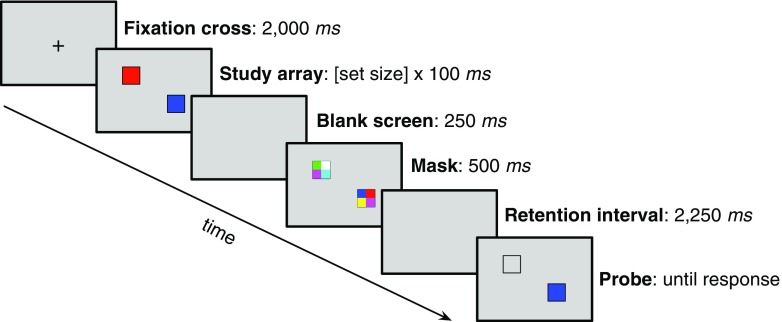
A schematic representation of a set size two trial in the simultaneous presentation condition. Note that the image is not to scale

The overall structure of the task is depicted in Fig. 1. The trial started with a fixation cross that was on screen for 2,000 ms. The study time in the simultaneous block was a linear function of the set size (study time = set size × 100 ms) and the set sizes were 2, 4, and 8. We adopted this deviation from the more typical visual change detection task in which the timing of the stimulus display is constant in order to ensure that exposure time to the objects was constant across the simultaneous/sequential manipulation. In the sequential block, the stimuli appeared one after another. Each stimulus was shown with a thin, black outline and remained on the screen for 100 ms. The stimulus color was then replaced with the background gray color and the black outline remained. After an inter-stimulus interval of 200 ms the following stimulus appeared on screen. The outlines of all stimuli remained on screen until a mask appeared. There was a 250 ms blank screen between the study array (or the final stimulus color in the sequential presentation) and the mask. The mask was displayed for 500 ms. Each individual stimulus mask was made up of a 4×4 grid of colored rectangles and the colors were randomly chosen from the same color set as the whole array. After the mask disappeared, a 2,250 ms retention interval (blank screen) delayed the onset of a single probe. The probe remained on screen until the participant responded. Alongside the probe were thin, black outlines of the other stimuli from the study array, which were displayed to prevent the participant from being unsure about which of the studied stimuli was probed.

## Results

**Fig. 2:**
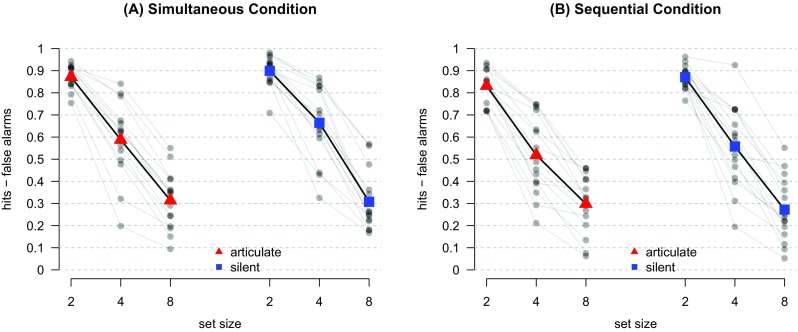
Descriptive statistics for the relevant performance measure *d* across the different conditions of the experiment. *Semi-transparent black circles* show the mean performance in each condition per participant and lines connect individual participants’ means. *Larger, colored symbols* are group means for each condition, connected by *thicker, black lines*

Prior to data analysis, all trials containing invalid responses (0.1 % of trials) were removed, and trials with unusually long or short response times ( <200 ms or >3 s; 2 % of trials) were excluded. The overwhelming majority of these were too slow, possibly because participants took unscheduled breaks by deliberately delaying their response. Overall, 36,495 trials across the 15 participants remained for analysis. Descriptive statistics for task performance across conditions are summarized in Fig. 2. Overall accuracy is high in the set size 2 condition, as expected, and decreases as set size increases. In addition, Table 1 shows the mean hit and false alarm rates across all participants.

**Table 1:** Mean hit and false alarm rates for all conditions across all participants

	hits			
	simultaneous		sequential	
set size	articulate	silent	articulate	silent
2	0.95 (0.022)	0.95 (0.036)	0.94 (0.026)	0.94 (0.032)
4	0.84 (0.065)	0.88 (0.063)	0.82 (0.076)	0.82 (0.097)
8	0.72 (0.079)	0.70 (0.100)	0.70 (0.101)	0.70 (0.174)
	false alarms			
2	0.08 (0.040)	0.06 (0.035)	0.11 (0.066)	0.07 (0.039)
4	0.25 (0.131)	0.22 (0.132)	0.31 (0.166)	0.26 (0.154)
8	0.41 (0.120)	0.39 (0.138)	0.41 (0.142)	0.42 (0.159)

Numbers in parentheses are standard deviations of the corresponding means

In order to assess the performance while controlling for response bias, for each condition-participant-set size combination we subtracted the false alarm rate from the hit rate to form an overall performance measure *d* (Cowan et al. 2005; Rouder et al. 2011). Of particular interest is how the performance advantage for the silent condition is affected by the type of presentation. If participants verbalize when the presentation is sequential, we would predict that articulation would hurt performance more with sequential presentation, and thus the advantage for the silent condition would be larger with sequential presentation.

**Fig. 3:**
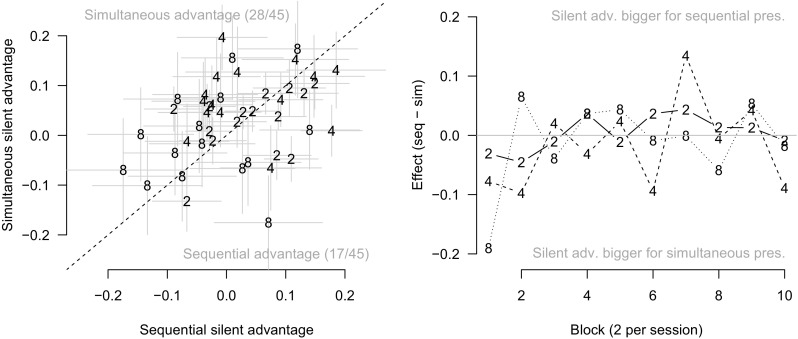
**a**: Advantage for silent condition (i.e., *d* in silent condition minus *d* in articulate condition) with simultaneous presentation as a function of the same for sequential presentation. Each point represents a single participant and set size. *Error bars* are approximate standard errors. **b**: The difference between the advantage for the silent condition in the sequential and simultaneous presentation conditions as a function of experimental block. In both plots, the number for each point represents the set size

Figure 3a plots the silent advantage in the simultaneous condition as a function of the same for the sequential condition for all participant by set size combinations. If participants were verbalizing, then being silent should aid performance. Moreover, being silent should aid performance *more* in the sequential-presentation condition than in the simultaneous-presentation condition. This prediction would appear in Fig. 3a as points falling below the diagonal. However, 28 out of the 45 points actually fall *above* the diagonal, inconsistent with the verbalization hypothesis.

It is plausible to suppose that participants only sometimes engage in verbal recoding, perhaps when it is most natural, or when they believe it will be most helpful (e.g., Larsen & Baddeley, 2003). Larsen and Baddeley surmised that participants abandon articulatory rehearsal with long or otherwise difficult-to-rehearse verbal lists. Building on this assumption, one might imagine that participants engage in strategic verbal recoding for small set sizes where helpful, distinct labels may be generated for each item, but abandon this strategy for larger set sizes. However, for all set sizes, the number of points above the diagonal in Fig. 3a is greater than one-half: 8/15, 10/15, and 10/15 points lie above the diagonal for set sizes 2, 4, and 8, respectively. There is no evidence of the predicted effect in these data; instead, the effect appears to go in the wrong direction.

We also examined whether the apparent lack of an effect may be due to differences in strategy over the experimental sessions; however, a similar picture emerges when the effect is examined across time, as in Fig. 3b. The verbalization hypothesis would predict that points would fall above the horizontal line at 0 on average; however, if anything, the points tend to fall *below* the line.

Given the descriptive analysis above, we eschew typical ANOVA analyses in favor of reliance on a state-trace analysis.^1^ We have the luxury of avoiding the assumption-laden ANOVA because we have directional predictions that are violated in the data. Thus, there cannot be evidence for the prediction of interest. Furthermore, we are interested in the dimensionality of the latent system that has produced the observed data - a question that an ANOVA, unlike state-trace analysis (Prince et al. 2012), cannot provide a reliable answer to. The state-trace analysis complements the descriptive analysis by showing that the data are highly consistent with a simple explanation: that performance is governed by a single latent variable (interpreted as visual short term memory capacity) and no more complicated explanation involving verbalization is needed.

### State-trace analysis

Another way to examine whether there is any evidence for verbalization is a *state-trace analysis*. State-trace analysis, outlined in its original form by Bamber (1979), is a data analysis technique intended to reveal how many latent dimensions a system requires to produce observed empirical results (see Prince et al., 2012 for an overview and the application of Bayesian analysis). A simple system may have only one latent dimension (e.g., working memory capacity in general, or visual working memory capacity specifically), and all experimental manipulations affect performance along that latent dimension. More complex systems may show relationships that are impossible to explain by a single dimension, and therefore require positing more latent constructs (see section *Diagnosing Dimensionality* in Prince et al. (2012) for a detailed explanation based on hypothetical examples).

Considering visual change detection performance, one might imagine that only one latent memory dimension contributes to recognition accuracy or alternatively that separate visual and verbal memory systems jointly contribute to recognition accuracy. The multi-component model of working memory (Baddeley 1986) proposes sub-systems for verbal and visual short-term memory, and would be consistent with the suggestion that both verbal and visual codes are stored during visual array memory, with both codes contributing to recognition accuracy. This assumption is the reason why precautionary articulatory suppression is so often employed during visual memory tasks. One reasonable prediction of the multi-component model is thus that at least two latent factors, verbal and visual memory, contribute to visual change recognition accuracy. Another reasonable expectation is that whether or not verbal encoding occurs, it is insufficient to affect recognition accuracy in this task, and in that case, a single dimension would better explain recognition accuracy in visual change detection. If visual change detection performance in our study, which was explicitly designed to allow verbalization to exert effects in specific conditions, can be explained by a single latent dimension then we would conclude that articulatory suppression is not needed to prevent verbalization in tasks with similar designs.

In the logic of state-trace analysis, performance in the sequential and simultaneous presentation conditions arise from either one or more latent constructs. If they both arise from a single latent variable, such as (visual) working memory capacity — and if performance in both is a monotone function of the latent variable – then performance in the sequential presentation must be a monotone function of performance in the simultaneous condition. To the extent that no monotone function can describe the relationship between simultaneous and sequential task performance, two latent constructs — perhaps distinct visual and verbal working memory capacities — are assumed to be needed to describe the performance.

For the state-trace analysis, we again used *d*, the hit rate minus the false alarm rate, as a measure of performance in our simulations. To reduce possibly spurious deviations in our simulations, we computed Bayesian estimates of *d* applying three reasonable constraints: first, we assumed that the true hit rate was greater than the true false alarm rate, and thus performance was truly above chance. Second, for both the sequential and the simultaneous condition, *d* must decrease with increasing array set size; for instance, true *d* to a set size of 8 cannot be better than performance to set size 4, all other things being equal. Third, it was assumed that suppression cannot benefit performance; for each set size and presentation condition, the true *d* in the articulate condition must be less than in the silent condition. This restriction was applied because a small dual-task cost appearing in all conditions would be consistent with any working memory theory, and with our distinctly-colored stimuli and meaningless articulation instructions, no benefit of articulation was reasonably expected. When a simulation produced one of these patterns, we excluded it and replaced it. Estimating the true discrimination under these restrictions yields a less error-prone measure of performance due to the exclusion of simulations with implausible data patterns.

**Fig. 4:**
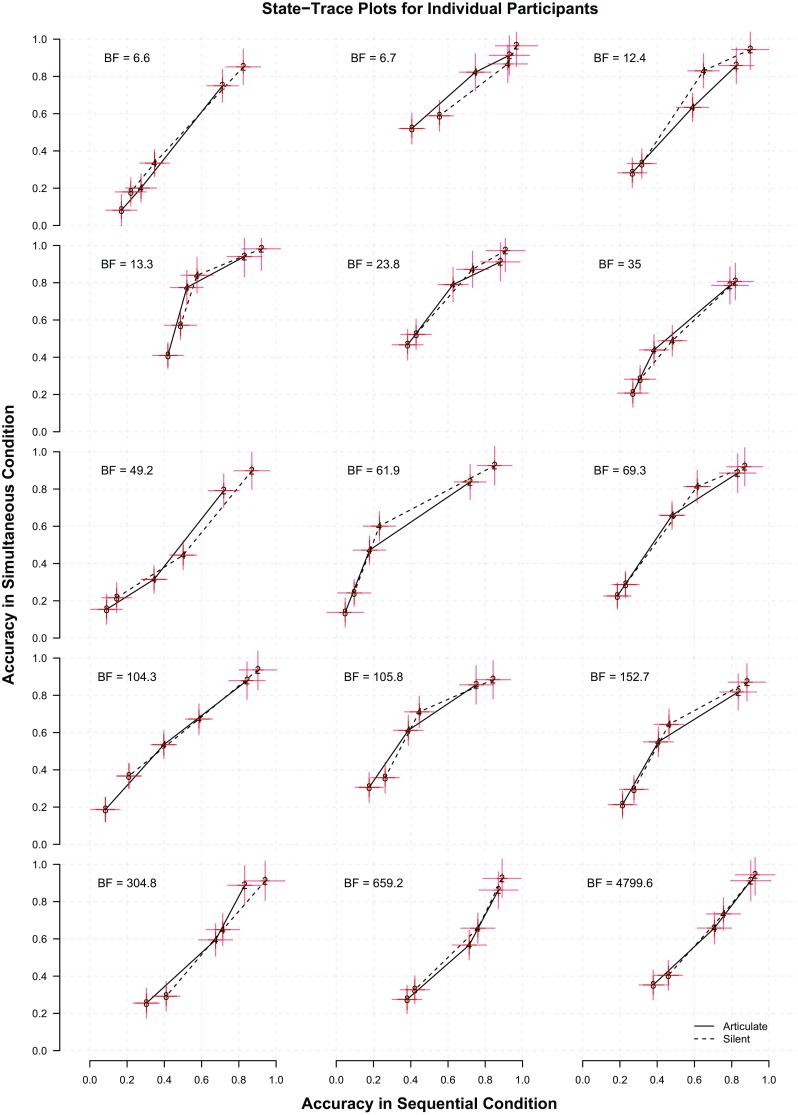
Individual state-trace plots for all 15 participants. The dependent variables are hit rate minus false alarm rate *d* for the three set sizes (2, 4, and 8) and are plotted with standard errors. In the *top left corner*, each plot also features the Bayes factor in favor of a monotone ordering of the points over a non-monotone ordering

Figure 4 shows the state-trace plots for each participant, formed by plotting estimated performance in the simultaneous presentation condition against the performance in the sequential condition. State-trace logic says that more than one latent construct is needed to explain the data when these points cannot be joined by a single, monotone curve; however, as can be seen from the state-trace plots for all participants, the state-trace plots are strikingly monotone. There does not appear to be any evidence that more than a single latent construct — (visual) working memory capacity — is needed, and thus no evidence that verbalization plays a role in performance in this task.

To quantify the support for monotonicity in the state-trace plots, we computed Bayes factors comparing the evidence for two hypotheses: first, that the true performance underlying the state-trace plots are ordered the same on both axes (that is, they can be described by a monotone curve), and second, that they are not ordered the same on both axes (Prince et al. 2012). We refer the reader to Prince et al. (2012) for technical details, and to the supplement to this article for details of how these Bayes factors were computed (see also Davis-Stober, Morey, Gretton, & Heathcote, 2015).

In addition to the state-trace plots for each participant, Fig. 4 also contains the Bayes factor favoring a monotone ordering of the points over a non-monotone one. The Bayes factors uniformly favored the monotone ordering of the points. The Bayes factors ranged from about 7 to almost 5,000. These data do not appear to provide any evidence for a deviation from monotonicity. Because our manipulations were designed to introduce effects of articulation consistent with the notion that verbal labeling can occur during visual memory tasks and can sometimes aid performance, this persistent monotonicity suggests that, at least for paradigms like this one, verbal labeling does not contribute to visual change detection performance.

## Discussion and Conclusions

The main question motivating the experiment we report was whether verbalization assists with other processes to influence visual memory performance. In that case, the application of articulatory suppression would be required to disengage a verbal memory dimension so that a pure measure of visual memory performance could be obtained. Neither a straightforward descriptive analysis nor a state-trace analysis revealed evidence that participants engaged in verbalization or that verbalization helped visual recognition memory, despite the fact that the experimental design favored the use of verbalization even more than the typical design of visual change detection tasks. The absence of a complex relationship between suppression, presentation type, and performance provides evidence that verbal recoding was not a strategy adopted by the participants in this task. Unlike previous studies which did not show effects of articulation on visual change detection performance, we were able to quantify evidence in favor of the null hypothesis for each individual participant using Bayesian state-trace analysis, providing novel positive evidence for the absence of this effect.

These results do not rule out any particular model of working memory. One interpretation of the multi-component working memory model (Baddeley 1986), namely that both verbal and visual codes would be generated and maintained during visual change detection tasks, was unsupported by our analysis. The assumption that verbal codes could be generated during visual change detection is not a proposal of the model, but merely an assumption made by researchers that is consistent with the model. Verbal encoding of visual materials is not necessarily obligatory. However, our results do have important practical implications for researchers interested in measuring visual working memory capacity. Our analyses confirm that for briefly-presented, abstract visual materials whose to-be-remembered elements are not readily encompassed by a verbal label, verbal labeling either does not occur at all, or if it does occur, does not contribute to recognition accuracy. These results are not inconsistent with the multi-component working memory model, but suggest that it is not reasonable to invoke this influential model to support arguments that verbal encoding of visual materials necessarily contaminates estimates of visual working memory capacity.

Another possible interpretation of the multi-component model of working memory is that the central executive component, which directs attention within the system, may only be applied to a single sub-system at once. This supposition might lead to predictions that individuals strategically choose to encode visual materials in verbal code or alternatively in visual code. Though it would be difficult to eliminate such a flexible account of the encoding of visual materials entirely, we think that our data tend to rule out this idea as applied to visual change detection. If this strategic choice of coding occurred, then it might reasonably have occurred only in the sequential condition, or only for small set sizes, or might have been especially prevalent in the sequential conditions for small set sizes. Evidence against the interactions that would support these predictions are provided by descriptive analysis: for all set sizes, more participants showed a greater silent advantage in the simultaneous condition, contrary to predictions. Note that the multi-component working memory model generates no explicit prediction that participants must strategically switch between encoding materials in verbal or visual code; indeed, it has been shown that encoding verbal and visual-spatial stimuli can proceed with little if any dual-task cost (Cowan and Morey 2007; Morey et al. 2013), which rather suggests that adopting a switching strategy would be unnecessary if one assumes that separate verbal and visual short-term memory sub-systems are available.

One caveat for the interpretation of these results is that state-trace analysis, like all methods, is limited by the resolution of the data. Detecting deviations from monotonicity in a curve depends on how finely points on the curve are measured. It is possible that with finer gradations of set size, we might be able to detect non-monotonicities that are not apparent in these data. However, visual inspection of the state-trace plots in Fig. 4 suggests that any effect of articulatory suppression is small; detecting such a small deviation from monotonicity would require finer gradations of sets size and more trials per set size. Our design already included thousands of trials per participant, and detected no positive effect of articulation condition while providing robust positive evidence for monotonicity. Even if a small deviation from monotonicity existed, it would be unlikely to have any substantial effect on measurements of visual working memory capacity.

In some instances, verbalization clearly effects visual memory performance (e.g. Brandimonte et al. 1992), but features of the stimuli and the task likely limit the potential effects of verbalization. Stimulus presentation duration is likely a crucial factor determining whether verbalization strategies are employed in visual memory tasks. The abstractness of the stimuli employed likely also influences the extent to which verbalization occurs. In instances in which verbalization appeared to assist visual memory, abstract visual patterns were shown for 3 seconds, with retention intervals of 10 seconds, allowing plenty of time for both the generation and rehearsal of verbal labels (Brown and Wesley 2013; Brown et al. 2006). Moreover, in each of these studies demonstrating effects of verbalization, stimuli that were amenable to verbalization (determined by pilot testing) were chosen. In an investigation of effects of articulation on color-shape memory in which only articulation of visually imaginable phrases harmed visual recognition, participants were given 4 seconds, one second per visual object, to study the objects for a later memory test (Mate et al. 2012). In contrast, the stimulus presentation timings we employed (100–300 ms per item, depending on whether inter-stimulus intervals are considered) were substantially faster than those used in paradigms meant to encourage verbalization, and our stimuli were random patterns of colors. Recognition of the color and its spatial location was required to respond correctly. These design features are representative of visual change detection paradigms generally. The timings we chose are within the range of the visual change detection papers cited in our Introduction, which range from 8 ms per item (Woodman and Vogel 2005) to as much as 500 ms per item (Brockmole et al. 2008). We conclude that for presentations as fast or faster than the 100 ms per item rate that we measured, it appears safe to assume that verbalization does not augment visual change detection performance.

Researchers employing nameable visual stimuli at paces enabling verbalization should still consider employing precautionary articulatory suppression if their goal is to isolate visual memory specifically. However, based on our data, we conclude that for many typical visual memory paradigms, such as those using brief presentations of randomly-generated abstract images, this precaution is unnecessary. Enforcing precautionary articulatory suppression does not seem to be necessary to get interpretable data from visual change detection tasks.

## Footnotes

1 For those interested, a traditional repeated measures ANOVA has been included in the online supplement available at https://github.com/fsense/opportunity-verbalization-statetrace
